# Evaluation of miR-141, miR-200c, miR-30b Expression and Clinicopathological Features of Bladder Cancer

**Published:** 2015

**Authors:** Ali Mahdavinezhad, Seyed Habibollah Mousavi-Bahar, Jalal Poorolajal, Reza Yadegarazari, Mohammad Jafari, Nooshin Shabab, Massoud Saidijam

**Affiliations:** 1 *Research Center for Molecular Medicine, Hamadan University of Medical Sciences, Hamadan, Iran.*; 2 *Urology and Nephrology Research Center, Hamadan University of Medical Sciences, Hamadan, Iran.*; 3 *Research Center for Modeling of Non-communicable Diseases, Department of Epidemiology and Biostatistics, School of Public Health, Hamadan University of Medical Sciences, Hamadan, Iran.*; 4 *Department of Pathology, Medical School, Hamadan University of Medical Sciences, Hamadan, Iran.*

**Keywords:** Urinary bladder neoplasm, microRNA-200c, microRNA-141, microRNA-30b, neoplasm grading, neoplasm invasiveness

## Abstract

Bladder cancer (BC) ranks the second most common genitourinary tract malignant tumor with high mortality and 70% recurrence rate worldwide. MiRNAs expression has noticeable role in bladder tumorigenesis. The purpose of this study was to assess miR-200c, miR-30b and miR-141 in tissue samples of patients with BC and healthy adjacent tissue samples and their association with muscle invasion, grade and the size of the tumor. Transurethral resection tissue samples were collected from thirty- five newly diagnosed untreated patients with BC from 2013 to 2014. The control group consisted of adjacent normal urothelium. All samples, observed by two pathologists, were diagnosed transitional cell carcinomas (TCC) with the proportion of tumor cells greater than 80%. Total RNA including miRNAs was extracted from about 50 mg tissue samples by applying TRIzol reagent. 2^(-ΔΔ CT)^ method was used to calculate relative quantiﬁcation of miRNA expression. Two of 35 patients were females and the other 33 were males. Invasion to bladder muscle was observed in 13 (37%) cases. MiR-141, miR-200-c and miR30-b were up-regulated in 91%, 79% and 64% of malignant tissues, respectively. Down-regulation of miR-141 had a strong association with muscle invasion (P= 0.017). Significant inverse correlation between grading and miRNA-141 level was observed (P= 0.043).

Bladder cancer (BC) ranks the second most common genitourinary tract malignant tumor with high mortality and 70% recurrence rate worldwide (1-2). The fourth most common cancer is allocated to BC in Western industrialized countries (2-3) and it is among the top ten malignancies which leads to death (4). Based on invasiveness to muscle, BC is divided into non-muscle invasive BC (NMIBC) and muscle-invasive BC (MIBC). The latter being about three times more prevalent (5).

Despite unknown exact mechanism of BC, irreversible genetic and reversible epigenetic changes, chromosomal anomalies and genetic polymorphisms are involved in tumorigenesis and progression of BC. Genetic changes are noticeable in BC prognosis and treatment (6-8).

MiRNAs are a class of small non- coding RNA molecules, about 22 (18-25) nucleotides, which negatively modulate gene and protein expression. MiRNAs and alteration in their expression may play a critical role in initiation, differentiation and development of various human malignant tumors as oncogenes or tumor suppressors (1, 3, 9-12). 

MiRNAs regulate translation of more than one-third of human mRNA species and protein-coding genes (12, 13), nevertheless; miRNAs impression in gene regulation has not been clarified completely (8).

Different miRNAs expression between tumor and normal tissues could explore the role of miRNAs involved in carcinogenesis, which in turn, may result in discovering novel therapeutic, diagnostic and prognostic markers (13). Alteration in miRNA expression may occur earlier in BC and affect carcinogenesis and tumor behavior (14). Gottardo et al. firstly reported 10 up-regulated miRNAs in BC (15). Altered expression of many miRNAs have been identified in Dyrskjot et al.'s study (16). Some miRNAs are related to epithelial- mesenchymal transition (17-19). Therefore miRNAs expression has noticeable role in bladder tumorigenesis.

Some researchers have reported overexpres-sion of miR-200c and miR-141 (20- 24) in BC while others reported underexpression of miR -141, miR -200c and miR 30b in BC (19, 25).

Among these miRNAs, in a study, 3 miRNAs panel, miR-200c, miR-141 and miR-30b, have displayed a sensitivity of 100% and a speciﬁcity of 96.2% to distinguish invasive BC from non-invasive BC (19).

Cystoscopy and histopathological analysis of biopsy specimens is the cornerstone of early diagnosis as well as the evaluation of prognosis of BC. Prognosticators such as tumor grade, stage, size, and multifocality can not predict clinical outcome accurately (26).

Thus, miRNAs, similar to their target proteins, are supposed to be useful as new biomarkers to improve the diagnosis and prognosis of different BC (14, 19, 27).

To our knowledge, miRNAs expression between NMIBC and MIBC as well as low grade versus high grade tumors have been compared in few literatures (18, 28, 29).

The purpose of this study was to assess miR- 200c, miR- 30b and miR- 141 in tissue samples of patients with BC and healthy adjacent tissue samples and their association with muscle invasion, grade and the size of the tumor.

## Material and methods

**Clinical sample collection**

All eligible patients with BC, diagnosed clinically and confirmed pathologically, irrespective of sex and age were included in this study. If patients had one of the following criteria, they were excluded from the study:(a) other organ or genitourinary cancers, (b) genitourinary infection, (c) history of radiotherapy or chemotherapy.

Transurethral resection tissue samples were collected from thirty-five newly diagnosed untreated patients with BC at shahid Beheshti and Bu-ali hospitals (Hamadan, Iran) from 2013 to 2014. Control samples consisted of adjacent normal urothelium resected about 10 cm far from the tumor lesion. Cystoscopy and clear description of tumor number, extent, location and type was done by an expert urologist under spinal anesthesia. The entire tumor as deep as needed and possible, was resected. After complete tumor resection, bladder and urethra were washed three times with normal saline, then a normal urothelium area sample was taken at least 10 cm far from tumor bed. The samples were washed with RNase-free cold saline solution immediately after resection and snap-frozen in liquid nitrogen. All samples were stored at -80 ºC until pathologic confirmation and further analysis (24). We also used a pre-determined questionnaire consisting of demographic characteristics (sex, age, smoking, exposure to carcinogens and living region) and imaging and pathological findings (tumor size, grade, muscle invasion, tumor type) to collect patients’ data.

Study protocol approval was done by the Ethics Committee of Hamadan University of Medical Sciences and Health (Hamadan, Iran). After explaining the aim of the study, written informed consent was taken from the chosen patients. The study protocol conforms to the ethical guidelines of the 1975 Declaration of Helsinki.

**Pathological confirmation and findings**

All samples, observed by two pathologists, were diagnosed transitional cell carcinomas (TCC) with the proportion of tumor cells greater than 80% (24). The 1973 World Health Organization criteria applied to assess tumor grading.

**Total RNA Extraction**

Total RNA including miRNAs was isolated from about 50 mg tissue samples by applying TRIZOL reagent (Invitrogen, Germany) according to the manufacturer's protocol. The extracted RNA was dissolved in 20- 50 µl RNase- free water depending on the quantity of the precipitation. Determination of RNA concentration and purity was performed by optical density measurement using a Nano-Drop spectrophotometer (Bio-TeK, USA). Integrity and quality of RNA were evaluated by 1% agarose gel electrophoresis.

**Real-time quantitative PCR analysis**

MicroRNA RT-PCR system (PARSGENOME, Iran) was used to determine microRNA expression by RT-qPCR. Briefly, 2 µg of RNA was reversely transcribed using specific primers according to the manufacturer's instructions. Afterwards, Real-time PCR amplification with SYBR Green master mix and miR specific primers completed by fifty ng of synthesized cDNA in a CFX96 real- time PCR detection system (Bio-Rad, USA) in duplicate. Melting curve analysis was performed to evaluate the specificity of primers.

5s rRNA as reference gene was used to normalize Ct values because of non- differential expression level in tumor and healthy adjacent samples. No template control (NTC) was included in each PCR run to evaluate contamination which were all negative. We used 2^(-ΔΔ CT)^ method to calculate relative quantiﬁcation of miRNA expression (30). Findings greater and less than 1 were determined to classify up-regulation and down- regulation, respectively. ΔΔCT formula is as follows: ΔΔCT= ΔCT_1 _– ΔCT_2_, which ΔCT_1_ = CT of the miRNA target (tumor sample)– CT of the reference gene (tumor sample) and ΔCT_2_= CT of miRNA target (normal tissue sample)-CT of reference gene (normal tissue sample).

**Statistical analyzes**

Statistical analysis was performed using STATA- 11 (StataCorp, College Station, TX, USA) software. All values were reported as mean± SD. ANOVA and t-test were used to compare grade, muscle invasion and size of tumor. A p-value less than 0.05 was considered significant.

## Results

Seventy tissue samples from 35 patients with BC were included in this study (35 from malignant region and 35 from adjacent normal tissue). In processing stage, two RNA samples of each group were excluded because of RNA degradation; therefore, 66 specimens were studied. All cancers were diagnosed as TCC. Two in 35 patients were females and the remaining 33 were males. The mean age of patients was 71.06± 11.43 (a range of 44 to 91 years old). In pathological assessment, invasion to bladder muscle was observed in 13 (37%) cases categorized as MIBC, the rest was NMIBC. Clinico-pathological characteristics inclu-ding sex, smoking, grade, region and muscle invasion of all patients have been illustrated in table 1 in details.

The mean CT values of 5s rRNA in case and control group (case: 14.93± 2.05 and control: 16.07± 3.19, p= 0.084) and between MIBC and NMIBC group (15.30± 2.30 and 14.72± 1.92, p= 0.448, respectively) was similar as well as among different gradings (G1: 13.94± 0.69, G2: 15.03± 2.11, G3: 14.93± 2.16, p= 0.788); therefore, it was a suitable choice as reference gene to normalize gene expression between different groups in this study. The miRNA primers were specific because a single peak was observed on melting curve analysis.

It was observed that miR-141 was up-regulated in 91% of malignant tissue samples. MiR-200-c and miR30-b were over-expressed in 79% and 64% of case group, respectively (Table 2).

There was no significant correlation between miR-30b, miR-141 and miR-200c expression and smoking state. In smokers, miR-30b expression was 5.42± 1.81 and in non– smokers, was 4.70± 1.96, p value= 0.292. In smokers, miR-141 expression was 1.50± 2.62 and in non-smokers was 1.33± 2.49, p value= 0.854. MiR-200c expression in smokers and non- smokers were as follow: -0.36± 2.32 and -0.59± 2.80, p value= 0.805.

**Table 1:** Clinico- pathological characteristics of patients

**Frequences**	**Sex**		**Region**		**Smoking**		**Grade**			**Muscle invasion**	
	**Female**	**Male**	**Rural**	**Urban**	**Yes**	**No**	**G1**	**G2**	**G3**	**Yes**	**No**
Number	2	33	12	23	23	12	2	16	17	13	22
Percent	6	94	34	66	66	34	6	46	48	37	63

**Table 2:** Expression features of miR30-b, miR-141 and miR-200-c in malignant tissue samples

***Variable***	**Fold change ≤1**		**Fold change >1**		**Fold change >1.5**	
	**Number**	**Percent**	**Number**	**Percent**	**Number**	**Percent**
*miR-30b*	12	36%	21	64%	21	64%
* miR-141*	3	9%	30	91%	30	91%
*miR-200c*	7	21%	26	79%	24	73%

**Table 3:** ∆CT values (Mean ± SD) and relative quantification between NMIBC and MIBC

**Variables**	**NMIBC**			**MIBC**			**Difference**		**t-test**	**fold change in **MIBC **vs** NMIBC
	**Number**	**Mean**	**SD**	**Nmber**	**Mean**	**SD**	**Mean**	**SE**	**P value**	
Mean ∆CT30b	21	4.80	1.65	12	5.78	2.13	0.98	o.66	0.149	0.50
Mean ∆CT141	21	0.65	1.73	12	2.82	3.21	2.17	0.85	0.017	0.22
Mean ∆CT200c	21	-0.91	1.76	12	0.36	3.30	1.27	0.88	0.158	0.41

**Table 4:** Association between grading and miRNAs expression

	**Grade I**			**Grade II**			**Grade III**			**Anova**
**Variables**	**Number**	**Mean**	**SD**	**Nmber**	**Mean**	**SD**	**Number**	**Mean**	**SD**	**P value**
Mean ∆CT30b	2	5.43	0.30	15	4.53	1.84	16	5.72	1.88	0.208
Mean ∆CT141	2	1.24	1.39	15	0.29	1.77	16	2.55	2.87	0.043
Mean ∆CT200c	2	-0.46	0.68	15	-1.22	1.95	16	0.29	2.86	0.239

**Table 5:** Mean miRNAs expression according to tumor size

	**Size<10cm2**			**Size>10 cm2**			**difference**		**t-test**
**Variables**	**Number**	**Mean**	**SD**	**Nmber**	**Mean**	**SD**	**Mean**	**SE**	**P value**
Mean ∆CT30b	22	4.76	1.74	11	5.97	1.94	1.22	0.67	0.077
Mean ∆CT141	22	.94	2.18	11	2.45	3.04	1.51	0.92	0.109
Mean ∆CT200c	22	-1.02	2.22	11	0.71	2.62	1.73	0.87	0.054

The higher ∆CT means the lower expression; therefore, the expression level of miR-141 in MIBC group showed a statistically significant decrease compared to NMIBC (P= 0.017). The expression level of miR-30b and miR-200c in MIBC group was lower than that of miR-30b and miR-200c in NMIBC group. However, it was statistically non- significant. The mean, standard deviation and p-value of all miRNAs are presented in Table 3. As indicated in Table 3, down-regulation of miR-141 had a strong association with muscle invasion.

In tumor specimens, we observed significant inverse association between grading and miR-141 level (p=0.043). We also did not see any significant correlation between deregulation of miR-30b and miR-200c and grading (p= 0.208 and 0.239, respectively) (Table 4).

We also subdivided the tumor samples according to the size of tumors, calculated by ultra-sonography, into greater and less than 10 cm^2. ^Based on the extent of tumor, the expression of miR-30b and miR-200c was lower in tumor samples with a size> 10 cm^2^ compared to tumor samples with a size< 10 cm^2^. P- values, especially in miR-200c, were slightly above of significant level (P= 0.077 and 0.054, respectively) (Table 5).

## Discussion

BC is one of the most important and the second prevalent genitourinary tract cancer (1-2). It is important to screen and diagnose BC at the early stage and to predict recurrence and progression to enhance therapeutic response. As mentioned earlier, miRNAs play an important role in cancer initiation, progression and metastasis (31-32).

Studies have reported that miRNA play important roles in the pathophysiology of BC. MiRNA may contribute to tumorigenesis and help in molecular diagnosis, prognosis and targeted therapy (33-35). However, limited studies have investigated the exact function of miRNAs and their roles in BC and in clinic-pathological behaviors (18, 28-29). This study evaluated the association between tissue miR-141, miR-200c and miR-30b and clinic- pathological features of BC such as tumor size, grade and invasiveness.

Comparing the level of these miRNAs expression individually, over expression of miR-141, miR-200c and miR-30b was observed in 91%, 79% and 64% of cancer tissues, respectively. Ratert, XIE, Scheffer and Han studies showed up- regulation of miR-200c and miR-141 in BC compared to healthy control group (20, 21, 23, 24). However, these studies have not reported miRNA expression in details.

miR-30b, miR-141 and miR-200c expression in MIBC group was lower than NMIBC group. Down-regulation of miR-141 had a strong association with muscle invasion. Thus, it can be useful to predict BC progression. Wszolek et al. demonstrated that expression of miR-30b, miR-141 and miR-200c was reduced in invasive lesions of BC (19) which confirms our findings.

Compared to healthy tissue, in another study; miR-141 has been over expressed in malignant bladder tissue samples, while the expression level of miR-141 is lower in MIBC samples compared to NMIBC samples (24). Opposite to our results, Scheffer et al. have reported that serum’s miR-141 level is similar in BC patients and controls, as well as in patients with NMIBC and MIBC. They also concluded that there is no association between miR-141 expression and clinic-pathological parameters such as metastasis and grading. Therefore, the usefulness of serum’s miR-141 level in the diagnosis or prognosis of BC was not confirmed (21). Because of different studied samples, we can not get a precise conclusion, so it would be necessary to design a study consisting of paired serum and matching tumors to evaluate truly miRNAs expression.

Some literatures reported down-regulation (8) or up-regulation (15, 18) of miR-200c in BC compared to normal tissue and lower expression in invasive than superficial tumors (18, 19). Lee et al. proved a strong association between ZEB1 and miR-200c expression (22). ZEB1 and ZEB2, validated targets of miR-141 and miR-200c, contribute to epithelial- mesenchymal transition and cancer cell migration in cancer cell lines and ovarian cancer specimens (36). Despite of a controversy among different investigations, down- regulation of miR-141 and miR-200c in MIBC seems to be true in accordance with their target role in cancer invasion.

We observed lower expression of miR-30b and miR-200-c in tumor samples with a size> 10 cm^2^ compared to tumor samples with a size <10 cm^2^. A significant inverse correlation between grading and miR-141 level was confirmed in this study.

In advanced cancer, the miR-200 family (miR-200a, -200b, -200c, -141 and -429) are frequently silenced and play a role in epithelial to mesenchymal transition (18). In one research, miR-222 and miR-143 expression were correlated with tumor grade, size, carcinoma in situ, recurrence and progression (14). Some miRNAs were down-regulated in low- grade tumors and up- regulated in high- grade BC (8).

Further longitudinal studies need to be done about different miRNAs expression in BC to identify recurrence, progression, different stages of neoplasm, tumor stratification, clinical outcome, prognosis, disease-specific survival and overall survival (14).

MiR-200c, miR-141 and miR-30b are best classiﬁers of invasive from non-invasive BC and poor prognosis is linked to low level expression of these three miRNAs (19).

The study on three miRNAs panel (miR-200c, miR-141 and miR-30b) displayed sensitivity and speciﬁcity of 100% and 96.2%, respectively (19).

Different results of these kinds of studies may be due to different methods, regions, genetics and epigenetic alterations, lifestyles, study population, sample size, tumor grades and stages.

The follow-up of BC patients shows that down-regulation of miR-200c expression is related to disease progression to muscle invasive BC and poor prognosis. Therefore, miR-200c expression could be helpful in the prediction of BC progression and treatment decisions (18). However, we did not find significant association between miR-200c and muscle invasion.

In Song et al.'s study, miR-200c, miR-141, and miR-30b have the potential to diagnose invasive bladder tumors which were misdiagnosed in pathologic assessment of bladder biopsy specimens (13).

Discovering an effective diagnostic method for early detection, prevention, treatment and monitoring recurrence of BC is necessary (13, 37). Some cancer biomarkers such as miRNAs will be known and help in early diagnosis and prediction of the cancer prognosis. Therefore, further inves-tigations should be done about miRNA alterations in BC to understand real role of miRNAs in tumor initiation, progression, metastasis and treatment response.
